# Identification of Adiponectin Receptor Agonist Utilizing a Fluorescence Polarization Based High Throughput Assay

**DOI:** 10.1371/journal.pone.0063354

**Published:** 2013-05-14

**Authors:** Yiyi Sun, Zhihe Zang, Ling Zhong, Min Wu, Qing Su, Xiurong Gao, Wang Zan, Dong Lin, Yan Zhao, Zhonglin Zhang

**Affiliations:** 1 Chengdu Medical College, Pharmacy School, Chengdu, Sichuan Province, China; 2 Chengdu Lang-Guan Technology Co., Ltd., Chengdu, Sichuan Province, China; University of Oslo, Norway

## Abstract

Adiponectin, the adipose-derived hormone, plays an important role in the suppression of metabolic disorders that can result in type 2 diabetes, obesity, and atherosclerosis. It has been shown that up-regulation of adiponectin or adiponectin receptor has a number of therapeutic benefits. Given that it is hard to convert the full size adiponectin protein into a viable drug, adiponectin receptor agonists could be designed or identified using high-throughput screening. Here, we report on the development of a two-step screening process to identify adiponectin agonists. First step, we developed a high throughput screening assay based on fluorescence polarization to identify adiponectin ligands. The fluorescence polarization assay reported here could be adapted to screening against larger small molecular compound libraries. A natural product library containing 10,000 compounds was screened and **9** hits were selected for validation. These compounds have been taken for the second-step *in vitro* tests to confirm their agonistic activity. The most active adiponectin receptor 1 agonists are matairesinol, arctiin, (-)-arctigenin and gramine. The most active adiponectin receptor 2 agonists are parthenolide, taxifoliol, deoxyschizandrin, and syringin. These compounds may be useful drug candidates for hypoadiponectin related diseases.

## Introduction

Adiponectin, a 244-amino acid protein, is also known as Acrp30, AdipoQ, apM1 and GBP28 [1], [2]. Although adiponectin is synthesized mainly in adipose tissue (white adipose more so than brown adipose), it has also been detected at much lower concentrations in skeletal muscle, liver, colon, cardiac tissue, bone marrow, fetal tissue, salivary glands, placenta, cerebrospinal fluid, and breast milk [3]–[8]. Adiponectin contains four domains including an amino-terminal signal peptide, a species-specific variable domain, a collagen-like region of 22 Gly-X-Y repeats, and a carboxyl-terminal globular domain (g-Ad). Adiponectin exists in its full-length (f-Ad) and a shorter-length which corresponds to the g-Ad domain in human plasma that binds to the adiponectin receptors [1].

Adiponectin binds to a number of receptors including adiponectin receptor 1 (AdipoR1) and adiponectin receptor 2 (AdipoR2), as well as a cadherin-like receptor [1]. AdipoR1 shows high affinity for g-Ad and AdipoR2 shows intermediate affinity for g-Ad [9]. The expression of these receptor isoforms affects downstream signaling pathways differently- AdipoR1 mainly affects the AMP-activated protein kinase (AMPK) pathway and AdipoR2 mainly affects the peroxisome proliferator-activated receptor-alpha (PPARα) pathway-and this correlates to plasma insulin levels *in vivo* [10], [11]_ENREF_11. Depending on the experimental model, cytostatic/apoptotic effects of adiponectin can be associated with an increased activation of AMPK, reduced ERK1/2 signaling [12], inhibition of the Akt kinase and glycogen synthase kinase/β-catenin pathway [13], and/or enhanced expression of Bax and p53 pro-apoptotic genes [14]. It has been shown that decreased expression of AdipoR1/R2 diminishes adiponectin sensitivity and leads to insulin resistance [15]. It has also been shown that adiponectin has anti-inflammatory, and anti-atherogenic, pro-apoptotic, and anti-proliferative properties [1], [16]–[18]. Hypoadiponectinemia is associated with type 2 diabetes, insulin resistance, atherosclerosis, coronary heart disease, and malignancies [19]–[22]. These results strongly suggest that adiponectin and its receptors are potential intervention points for the development of anti-diabetes and/or anti-cancer agents.

Adiponectin-based therapeutics are not available currently, and since converting the full-size adiponectin protein into a viable drug has proven difficult, alternative compounds targeting the AdipoR could be designed through a peptidomimetic approach or identified using high-throughput screening against compound libraries [23]. As alternatives, specific and selective compounds targeting AdipoR could be designed through a peptidomimetic approach [23] or identified using high-throughput screening against compound libraries. However, these initial compounds are new, and their safety and toxicity, including potential long-term risks, would need to be determined. Given that the discovery, development and registration of novel drugs is a costly and very lengthy process (about 10–17 years), other efforts to develop adiponectin agonists merit consideration. Furthermore, natural products have long been used to treat diseases with efficacy and drug candidates derived from natural origins may mitigate adverse biological effects [24], [25]. Therefore, we conducted a high throughput screening against a natural compounds library and identified nine lead compounds. These compounds were validated in secondary *in vitro* assays and four of them, matairesinol, arctiin, (-)-arctigenin, and gramine show high activity in AdipoR1 system. Four of these hit compounds, parthenolide, taxifoliol, deoxyschizandrin, and syringing, showed high activity in AdipoR2 system.

## Materials and Methods

### Peptides and Proteins

Peptides (**Table 1**) were purchased from Pharmanic (Chengdu, Sichuan, China) with a purity ≥98%. The peptide concentrations were determined by amino acid analysis at the protein core facility (Chengdu Medical College). Recombinant g-Ad was produced in BL21(DE3) bacterial cells with a C-terminal His-tag. The full-length cDNAs encoding AdipoR1 and AdipoR2 were cloned, using pCR2.1–TOPO cloning kits (Invitrogen, China), from human cDNA libraries. The cDNAs encoding full-length of *AdipoR1* or *AdipoR2* were then sub-cloned into the mammalian expression vector pBEX and pcDNA3.1–Myc-His(+). His-tagged proteins were produced in mammalian cells (CHO) and purified using Ni-agarose beads, washed, and eluted from the Ni-agarose resin. Proteins were stored in 25 mM Tris.HCl, pH 7.3, 100 mM glycine, 10% glycerol. Proteins were subjected to dialysis against two changes of dialysis buffer (50 mM phosphate-buffered saline, pH 7.4) before experiment and the purity of the samples were tested by western blot of SDS-PAGE gel [13] (**Fig. S1**).

**Table 1:** Peptides used in FP assay.

Peptide	Sequence	Position of FITC label
Probe **1**	FITC-DAsn-Ile-Pro-Nva-Leu-Tyr-DSer-Phe-Ala-DSer-NH_2_	N-terminal
Probe **2**	H-DAsn-Ile-Pro-Nva-Leu-Tyr-DSer-Phe-Ala-DSer-FITC	C-terminal
Peptide **3**	H-DAsn-Ile-Pro-Nva-Leu-Tyr-DSer-Phe-Ala-DSer-NH_2_	No label

The primers used to clone full-length AdipoR1 and AdippoR2 cDNAs were:

AdipoR1:

Forward Primer: TGTCTTCCCACAAAGGATCTGT.

Reverse Primer: TCAGAGAAGGGTGTCATCAGT.

AdipoR2:

Forward Primer: GAACGAGCCAACAGAAAACCGA.

Reverse Primer: TCACAGTGCATCCTCTTCACTGC.

### Compound Library

The natural product library contains 10,000 natural products with a minimum of 98% purity confirmed by NMR and HPLC (Pharmanic, Chengdu, Sichuan, China). Compounds were present at 10 mM in DMSO. Compounds were seeded from A2 to H11.

### FP Assay Development

All fluorescence polarization measurements were made on 384-well, low-volume, black, round-bottom polystyrene microplates (Corning, Shanghai, China) using ZS-2 plate reader. The binding affinities of fluorescein-labeled peptides binding to unlabeled protein (AdipoR1 or AdiopR2) were verified by titrating an increasing concentration of protein into a constant concentration of labeled peptides. The polarization values were measured at an excitation wavelength of 485 nm and an emission wavelength of 538 nm. The total fluorescence (TF) values were also measured. The change in polarization was graphed as a function of the log of the protein concentration, and the dissociation constant (K_d_) was obtained from the resulting sigmoidal curve. The concentrations of fluorescein-labeled peptide as well as adiponectin receptors were optimized.

### FP Assay Stability Assessment

Unlabeled short peptide **3** was titrated against the FITC-labeled peptide and adiponectin receptor mixture. Plates were incubated at room temperature for up to 3 hours or 30 min at different temperatures. FP values were taken and IC_50_ values were calculated.

### DMSO Tolerance Assay

Increasing concentrations of DMSO at 1–20% of the assay volume (20 µl) were added to the mixture of FITC-labeled peptide and adiponectin receptor. FP and TF measurements were taken at room temperature (25°C) after 30 min incubation.

### FP Based Screen

Master mixtures containing fluorescein-labeled probe **1** and adiponectin receptor (AdipoR1 or AdiopR2, 2 µM and probe **1** at 100 nM) in a reaction volume of 19 µl were prepared and loaded to plates. The library compounds (10 µM –0.078 µM, half dilutions) were prepared separately and transferred into the assay plates. The first and last columns of each plate were used as positive and negative control wells. Wells containing the drug only were used as controls to detect auto-fluorescence of the compounds in the total fluorescence assay. After subtraction of the background signal, data was processed and the IC_50_ values were determined by non-linear least square fitting using SigmaPlot 11.0.

### Cell Culture

All cell lines were obtained from the American Type Culture Collection (ATCC). MCF-7 and MDA-MB-231 cells were maintained in DMEM containing 10% fetal bovine serum and supplied with 1% penicillin/streptomycin. All cells were maintained in a 5% CO_2_ atmosphere at 37°C.

### Drug Treatment for Signaling Pathway Analysis

Cells were grown to 80% confluence, harvested and aliquoted into 100 mm dishes in serum free media. Cells were allowed to attach overnight and then the serum free media was removed and then cells were washed in PBS twice. Different doses of compounds were prepared in the normal media and added to different dishes for an additional 6 h. Cells were washed 3 times with ice-cold PBS and cell lysates were prepared by scraping cells into lysis buffer A (RIPA buffer, 2 mM dithiothreitol (PMSF), 1×Halt protease inhibitor cocktail (Thermo scientific), and 1×Halt phosphatase inhibitor cocktail (Thermo scientific)). The cells were allowed to swell on ice (5 min), after which the homogenate was centrifuged. The supernatants were transferred to fresh tubes and protein concentrations were determined using the Bio-Rad protein assay (Bio-Rad Laboratories, Inc.) per manufacturer’s instructions. Aliquots of the cell lysates were stored at −80°C.

### Western Blotting

Equal amounts of total protein (50 µg) were subjected to electrophoresis (10% tris-HCl, 1.0 mm gels) and transferred to nitrocellulose membranes. Membranes were blocked in TBS containing 0.05% Tween 20 (TBST) and 5% nonfat milk or 5% BSA for 1 hour at room temperature. Appropriately diluted primary antibodies were incubated in TBST/5% nonfat milk or TBST/5% BSA for 2 h at room temperature or overnight at 4 °C. Membranes were washed in TBST three times for 15 min each. Appropriately diluted secondary antibodies were incubated in superblock blocking buffers (Thermo Scientific) for 1 h at room temperature. Membranes were washed in TBST three times for 15 min. Proteins were detected by Amersham™ ECL plus western blotting detection system (GE healthcare). The following antibodies were used: anti-AdipoR1 M18, anti-AdipoR2 C12 (Santa Cruz Biotechnology, Santa Cruz, CA), anti-p-AMPKα(T172), anti-AMPKα, and anti-β-actin (Cell Signaling, Danvers, MA).

### Cell Proliferation Assays

The effects of the lead compounds on cell proliferation were tested in MCF-7 and MDA-MB-231 cells. Cells were grown to 80% confluence, harvested and aliquoted into 384-well plates in a total volume of 45 µl per well. The outer wells were inoculated with sterile water to minimize evaporation from the sample wells. Cells were allowed to attach overnight and 5 µl culture media containing either vehicles or drug were added. Cell proliferation was evaluated using CellTilter-Blue reagent (Promega, China) according to manufacturer’s instructions after 72-h incubation. Fluorescence was measured with an excitation at 530 nm and emission at 590 nm. After background subtraction, cell viability values were normalized to vehicle controls and expressed as percentage of the mean of the relative vehicle controls.

### Statistical Analysis

Data were reported as mean ± SEM of 3–5 independent experiments, each treatment performed in duplicate or triplicate. Values were compared using the Student’s *t* test or with one-way ANOVA when three groups were present. Statistical significance was considered as *p*≤0.05.

## Results

### Screen Assay Development

The adiponectin active site has been identified during the development of a peptide-based adiponectin receptor agonist and a short peptide has been synthesized and tested as potential adiponectin receptor agonist [23]. In an attempt to identify AdipoR agonist, we developed a two-step screening process. The first step, a pilot screening, is a homogeneous FP-based assay to identify potential compounds that binds to adiponectin receptors. Fluorescein isothiocyanate (FITC) was used to label peptides derived from adiponectin globular domain (residues 105–204) which mimic the active site (**Table 1**) [23]. These fluorescein-labeled peptides bind to adiponectin receptors and can be used to identify compounds that competitively bind to adiponectin receptors.

**Figure 1:**
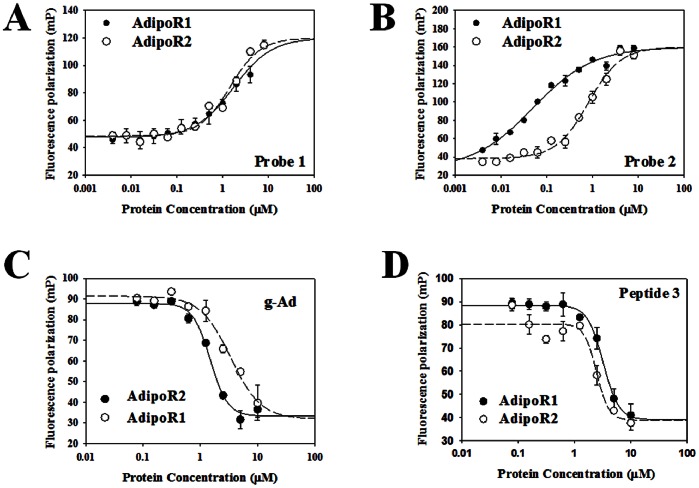
FP assay development. A and B, Adiponectin receptor – probe binding isotherms. An increasing concentration of protein (AdipoR1 or AdipoR2, 625 nM to 8 µM) was titrated into 100 nM probe (probe **1** or probe **2**). C and D, validation of positive control. Protein g-Ad or peptide **3** was titrated against the probe **1**– adiponectin receptor mixtures.

The FP assay utilizing fluorescein-labeled peptides binding to AdipoR1 or AdipoR2 proteins was developed and optimized in a 384-well format. Two probes were designed to determine the effect of FITC on the assay. Binding experiments were performed and K_d_ values were calculated. Titration of these probes with adiponectin receptors causes the FP and the fluorescence anisotropy (FA) to increase 2 to 3-fold. Binding affinities to probe 1 (**Fig. 1A**) were similar for AdipoR1 and AdipoR2- K_d_ = 1.72±0.62 µM and K_d_ = 1.45±0.23 µM, respectively. Probe **2** binds to AdipoR2 (K_d_ = 0.81±0.11 µM, **Fig. 1B**) with a higher affinity than AdipoR1 (K_d_ = 1.40±0.24 µM, **Fig. 1B**). Probe **2** has higher affinities for both adiponectin receptors. To study competitive inhibition using the FP binding assay, the concentration ratio between the receptors and the K_d_ of the labeled probes should be at least 1 in order to generate IC_50_ curve. Therefore, probe **1** was used as the probe for both adiponectin receptor systems. In addition, the receptor concentration needed for the FP assay should be ≥ K_d_. Therefore, 2 µM was used as the receptor concentration.

To determine whether probe **1** binds to the active site of adiponectin receptors and to choose a positive control, competition assays were carried out using g-Ad protein or peptide **3**. The addition of g-Ad was shown to cause the FP values returned to the level of free probes (**Fig. 1C**) which indicates the ability of g-Ad protein to replace probe **1**. Under these conditions, the IC_50_ values for g-Ad against AdipoR1 and AdipoR2 were 3.40±0.70 µM and 1.49±0.08 µM respectively (**Fig. 1C**). Compared to g-Ad, the ability of peptide **3** to compete with probe **1**– adiponectin receptor binding was slightly weaker (3.20±0.24 µM and 2.46±0.25 µM, respectively, **Fig. 1D**). Given that the present study is a screening study, peptide 3 was chosen as the positive control for cost-efficiency purposes.

**Figure 2:**
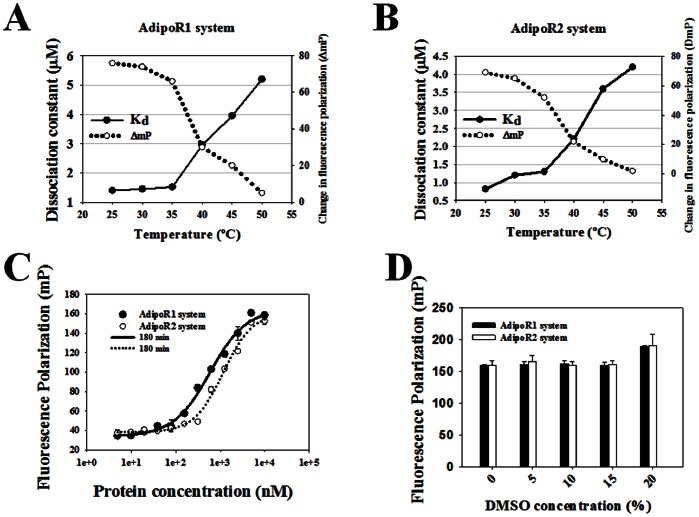
FP assay stability assessment. A and B, temperature dependence of K_d_ and ΔmP values. AdipoR1 or AdipoR2 was titrated against 100 nM probe **1**. C, assessment of stability of binding experiments over time. Adiponectin receptor was titrated against 100 nM probe **1**. FP values were measured at 25°C at 180 min. D, stability of binding experiments with increasing DMSO concentrations. DMSO was titrated into constant amounts of adiponectin receptors (2 µM) and probe **1** (100 nM). The total reaction volume was maintained constant in all wells at 20 µl. The measurements were made at room temperature (25°C) after 30 min of incubation.

To optimize the FP assay properly, it was necessary to assess the stability of the FP assay. The conditions for the FP assay were established by testing various parameters including incubation temperature, time, TF values, ΔmP values, and DMSO tolerance. Our data show that at room temperature (25°C) both receptor systems are stable. The K_d_ values increased and ΔmP values decreased when the plates were heated over 35°C, indicating the probe-receptor system started to dissociate (**Fig. 2A & B**). The incubation time was optimized to 30 min for rapid screening (**Fig. 2 B**) as we demonstrated the FP assay is stable over time. K_d_ = 1.76±0.22 µM (AdipoR1– probe **1**), and K_d_ = 1.40±0.09 µM (AdipoR2– probe **1**) (**Fig. 2C**). The DMSO tolerance for the FP assay was also tested, stable FP values (**Fig. 2D**) and TF values (data not shown) were observed at up to 15% DMSO in PBS buffer (v/v). In summary, the competitive FP binding assay conditions ideal for identifying adiponectin receptor agonists are: i) 2 µM adiponectin receptor, ii) 100 nM probe **1**, iii) 5% final DMSO concentration (v/v), and iv) incubation for 30 min at 25°C before reading the plate.

### Pilot Screen and Assay Performance Measurements

A pilot screen against the natural product library of 10,000 compounds described above was performed at screening concentrations of 10 µM to 0.078 µM (half serial dilutions) in 5% DMSO (v/v). Controls present in each assay plate consisted of 5% DMSO (negative control) or peptide **3** (positive control). The assay was semi-automated and the steps are listed in Table S1 ****.

The performance of the FP assay and post-screen analysis were summarized in **Table S2**. Assay performance was assessed using Z’ factor generated from whole DMSO plates.

### Post Screen Analysis

We screened a library of 10,000 natural compounds for adiponectin agonists. 954 compounds were excluded from the following post-screen validation if the compounds exhibited: (1) auto fluorescence, (2) quenching of fluorescence, (3) precipitation from assay, or (4) a decrease in polarization signal while lacking a dose response. 46 compounds that could generate IC_50_ curves were identified as initial hits. 9 candidate agonists that have an IC_50_ less than 5 µM (**Table 2**) were verified in more extensive competitive binding assays and their IC_50_ values were confirmed. The structures of the hit compounds are listed in **Figure S2**. Only one compound failed in the validation (chrysin) and was excluded. Interestingly, three compounds from the same plant, *arctium lappa,* all showed activity in the AdipoR1 system: arciin, (-)-Arctigenin, and matairesinol. Deoxyschizandrin, syringing, taxifoliol, and parthenolide showed activity in AdipoR2 system. Gramine showed activity in both systems.

**Table 2:** Summary of hit compounds.

Number	Compound	Derived from	IC_50_ vs AdipoR1 (µM)		IC_50_ vs AdipoR2 (µM)	
			Primary screen	Validated	Primary screen	Validated
**1**	Arctiin	Arctium lappa	1.2	1.3±0.2	>5.0	>5.0
**2**	(-)-Arctigenin	Arctim lappa	2.6	2.2±0.8	>5.0	>5.0
**3**	Matairesinol	Arctim lappa	0.9	0.8±0.2	>5.0	>5.0
**4**	Deoxyschizandrin	Schisandra berries P.E.	>5.0	>5.0	3.5	3.2±0.7
**5**	Syringin	Eleutherococcus senticosus	>5.0	>5.0	4.6	4.5±0.5
**6**	Taxifoliol	Chinese yew	>5.0	>5.0	3.5	3.0±0.3
**7**	Parthenolide	Tanacetum parthenium	>5.0	>5.0	1.5	1.2±0.1
**8**	Chrysin	Passiflora caerulea	>5.0	>5.0	4.9	>5.0
**9**	Gramine	Silver maple	4.5	4.2±0.5	3.6	3.2±0.2

### Effects of Hit Compounds on Adiponectin Receptor Signaling Pathways

**Figure 3:**
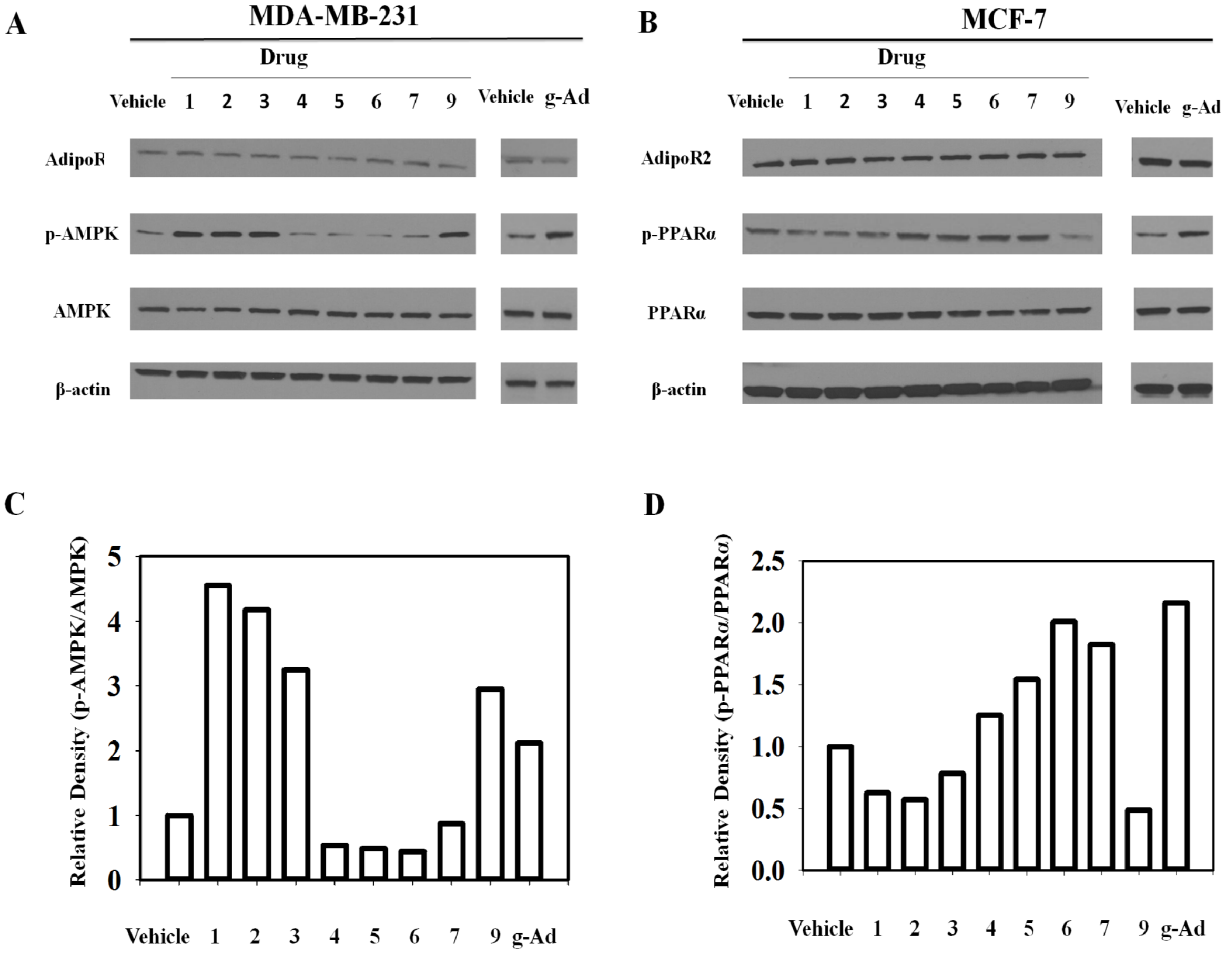
Effects of hit compounds on adiponectin receptor signaling pathways. A, MDA-MB-231 cells were treated with g-Ad or drugs at different concentrations (IC_50_) for 6 h. B, MCF-7 cells were treated with drugs at different concentrations (IC_50_) for 6 h. A total 50 µg of cell lysate was loaded to each lane. Western blot was performed follow the protocol in materials and methods section.

It has been demonstrated by several groups that human breast cancer cells including MDA-MB-231 and MCF-7 cells have detectable levels of adipoR1 and adipoR2 proteins. It has also been demonstrated that adiponectin has effects on proliferation of MDA-MB-231 and MCF-7 cells [14], [26]–[28]. Therefore, we chose this model to validate our screen hits. The second step is an *in vitro* screening using western blotting methods to further confirm the agonistic activity. It is well established that adiponectin activates AMPK and p38 MAPK through AdipoR1 or PPARα through AdipoR2 to mediate its downstream signaling cascades [8], [9], [12], [14]. IC_50_ values were obtained to further confirm the agonist activities of candidate compounds by treating MCF-7 or MDA-MB-231 cells with different concentrations of drugs or g-Ad for 6 hours. The levels of phosphorylation of AMPK and phosphorylation of PPARα were examined and the relative expression levels of the phospho-protein to the total expression of protein levels were quantified using ImageJ software (NIH) (**Fig. 3**). Compounds **1**, **2**, **3, 9** and the positive control g-Ad elevated AMPK phosphorylation in MDA-MB-231 treated cells while compounds **4**, **5**, and **6** decreased AMPK phosphorylation in MDA-MD-231 cells. Compound **7** has no effect on the AMPK phosphorylation level (**Fig. 3A**). Compounds **4**, 5, **6**, **7** and the positive control g-Ad increased PPARα phosphorylation level in MCF-7 treated cells. Compounds **1**, **2**, **3** and **9** inhibited p-PPARα level under the indicated condition (**Fig. 3B**).

### Hit Compounds Inhibit the Growth of AdipoR1/AdipoR2– Positive Cancer Cell Lines

To further confirm the agonistic activity of hit compounds, MDA-MB-231 and MCF-7 cells were treated with different concentrations of g-Ad or drug (20 µM to 1.2 nM) for 72 hours and cell viability was measured. Dose-dependent effects of these compounds were confirmed and IC_50_ values for cell growth are summarized in **Table** **3**. Thus, four compounds (arctiin, (-)-arctigenin, matairesinol and gramine) that show agonistic activity against adiponectin receptor 1 and four compounds (deoxyschizandrin, taxifoliol, syringing and parthenolide) that show agonistic activity against adiponectin receptor 2 were identified.

**Table 3:** Summary of cell viability assay results.

Number	Compound	IC_50_ (µM)	
		MDA-MB-231 cells	MCF-7 cells
**1**	Arctiin	3.2±0.3	>10
**2**	(-)-Arctigenin	4.5±0.4	>10
**3**	Matairesinol	1.8±0.6	>10
**4**	Deoxyschizandrin	>10	5.5±0.6
**5**	Syringin	>10	6.8±0.8
**6**	Taxifoliol	>10	4.4±0.5
**7**	Parthenolide	>10	2.5±0.4
**9**	Gramine	9.6±0.9	0.1±0.1
**10**	g-Ad	15.3±3.2	10.6±2.5

## Discussion

Design and development of adiponectin receptor agonist is an active field of study because adiponectin receptor agonists are being considered to treat hypoadiponectinemia that is associated with multiple diseases including obesity, diabetes and cancer [1], [8], [16]. Due to the difficulty of engineer adiponectin protein as a drug, it is important to search for existing agonists. FP is a solution-based, homogeneous technique requiring no immobilization or separation of reaction components and has been used to develop HTS assays. The principle of the screen is to identify the compounds that displace the probe **1** from adiponectin receptors by detecting the resulting decrease in FP. The fluorescent labeled peptide (probe **1**) bound to adiponectin receptors with a sub-micromolar dissociation constant was inhibited by g-Ad protein. Our data have proven this screen can be used as a fast robust, and quantitative for the determination of binding affinity for a wide range of adiponectin receptor agonists. The quantitative nature of the data significantly aids the efforts to choose the hit compounds. The Z’ factors of 0.68 and 0.64 indicate this assay could be easily adapted for high-throughput screening. We predict that this assay will be suitable for identifying small molecule agonists/antagonists of adiponectin receptors.

It is very interesting that our hit compounds (arctiin, (-)-arctigenin and matairesinol) are extracted from the same plant and show selective agonistic activity to AdipoR1. These compounds have been reported to have antioxidant and anti-proliferation properties [29], [30]. Deoxyschizandrin, Syringin, taxifoliol, parthenolide and gramine have been shown to have a wide range of biological activates including anti-viral, anti-inflammatory and anti-cancer properties [31]–[38]. These natural products have been long used in traditional Chinese medicine. However, the mechanisms of these drug actions are still largely unknown. In addition to demonstrating the utility of these screening methods with existing compounds, our data are novel in that they provide evidence that the effects of these compounds are due to the fact they are adiponectin receptor agonists. Additional novel findings include decreased p-PPARα in MCF-7 cells treated with gramine, previously posited to be a cytotoxic agent [37].

In all, here we report on the development of a fluorescence polarization based high-throughput assay to identify potential AdipoR agonists. Based on secondary assays, we have confirmed the agonistic activities of the hit compounds. These compounds may be considered as alternatives to a recombinant adiponectin protein for modifying AdipoR activity and could be studied in future experiments.

## Supporting Information

Figure S1 **Generation of recombinant g-Ad, AdipR1 and AdipoR2.** Recombinant g-Ad was produced in BL21(DE3) bacterial cells with a C-terminal His-tag. The full-length cDNAs encoding AdipoR1 and AdipoR2 were cloned, using pCR2.1–TOPO cloning kits (Invitrogen, China), from human and mouse testis cDNA libraries, respectively. The cDNAs encoding full-length of *AdipoR1* or *AdipoR2* were then sub-cloned into the mammalian expression vector pBEX and pcDNA3.1–Myc-His(+). His-tagged proteins were produced in mammalian cells (CHO cells) and purified using Ni-agarose beads, washed, and eluted from the Ni-agarose resin. Proteins were subjected to dialysis and the purity of the samples were tested by western blot of SDS-PAGE gel.
(TIFF)

Figure S2 **Chemical structures of hit compounds. A-I, drug 1–9.**
(TIFF)

Table S1 **Screen assay protocol.**
(DOCX)

Table S2 **FP assay performance and post-screen analysis summary.**
(DOCX)
