# Supplementary figures and images for: Identification of Adiponectin Receptor Agonist Utilizing a Fluorescence Polarization Based High Throughput Assay
Source: PLoS One. 2013 May 14;8(5):e63354. doi: 10.1371/journal.pone.0063354 (PMC3653934; doi:10.1371/journal.pone.0063354)

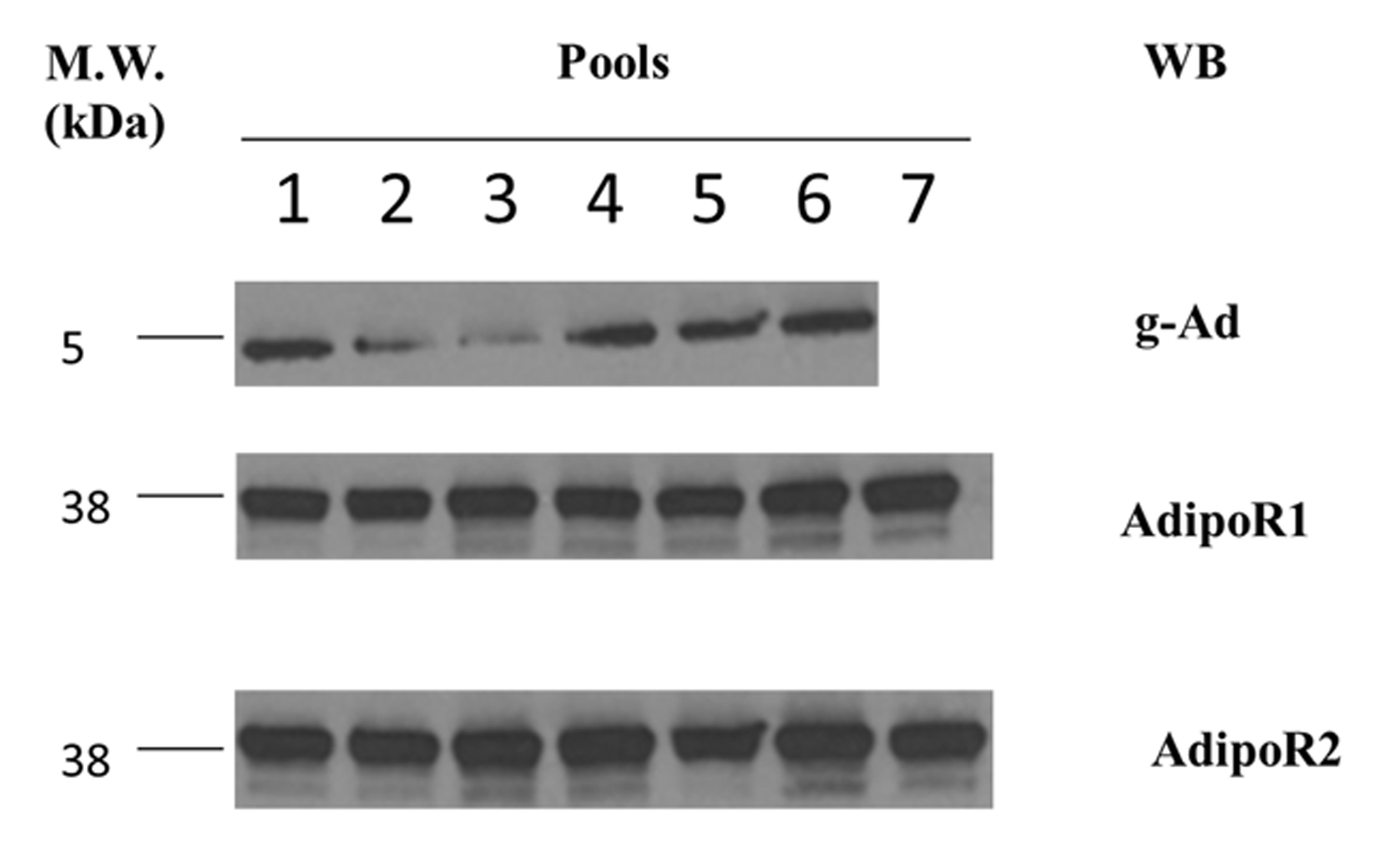

Supplement: Figure S1 — Generation of recombinant g-Ad, AdipR1 and AdipoR2. Recombinant g-Ad was produced in BL21(DE3) bacterial cells with a C-terminal His-tag. The full-length cDNAs encoding AdipoR1 and AdipoR2 were cloned, using pCR2.1–TOPO cloning kits (Invitrogen, China), from human and mouse testis cDNA libraries, respectively. The cDNAs encoding full-length of AdipoR1 or AdipoR2 were then sub-cloned into the mammalian expression vector pBEX and pcDNA3.1–Myc-His(+). His-tagged proteins were produced in mammalian cells (CHO cells) and purified using Ni-agarose beads, washed, and eluted from the Ni-agarose resin. Proteins were subjected to dialysis and the purity of the samples were tested by western blot of SDS-PAGE gel. (TIFF) [file pone.0063354.s001.tif]

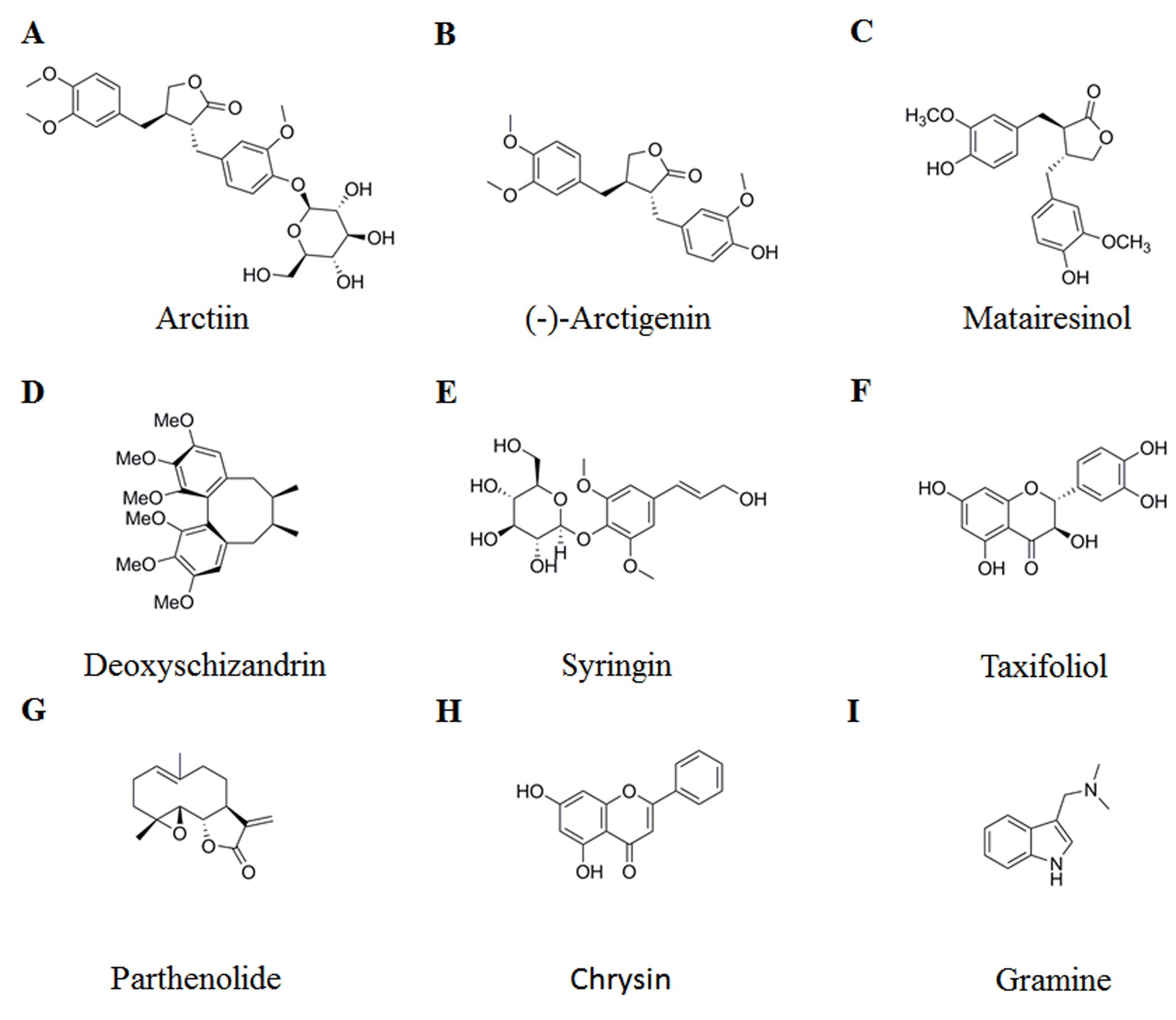

Supplement: Figure S2 — Chemical structures of hit compounds. A-I, drug 1–9. (TIFF) [file pone.0063354.s002.tif]
