# Supplementary material for: Identification of Adiponectin Receptor Agonist Utilizing a Fluorescence Polarization Based High Throughput Assay
Source: PLoS One. 2013 May 14;8(5):e63354. doi: 10.1371/journal.pone.0063354 (PMC3653934; doi:10.1371/journal.pone.0063354)
Supplement: Table S1 — Screen assay protocol. (DOCX) [file pone.0063354.s003.docx]

| Step | Parameter | Value | Description |
| --- | --- | --- | --- |
| 1 | Plate master mixture | 19 µl | Master mixture of AdipoR1 or AdipoR2 and probe **1** |
| 2 | Controls | 1 µl | 10 µM to 78 nM titration series of peptide 3 |
| 3 | Library compounds | 1 µl | 10 µM to 78 nM titration series |
| 4 | Incubate time | 30 min | Room temperature |
| 5 | Assay readout | Ex/Em=485/538 nm | ZS-2 plate reader |

Plates lidded until read.
