# Supplementary material for: Identification of Adiponectin Receptor Agonist Utilizing a Fluorescence Polarization Based High Throughput Assay
Source: PLoS One. 2013 May 14;8(5):e63354. doi: 10.1371/journal.pone.0063354 (PMC3653934; doi:10.1371/journal.pone.0063354)
Supplement: Table S2 — FP assay performance and post-screen analysis summary. (DOCX) [file pone.0063354.s004.docx]

| Category | Parameters | Descriptions |
| --- | --- | --- |
|  | Nature of the assay | Cell-free multicomponent competitive assay |
| Assay | Assay strategy | Detection of adiponectin receptor agonist using fluorescence polarization assay |
|  | Reagents and sources | See materials and Methods |
|  | Assay protocol | Key steps outlined in Table **2** |
|  | Nature of the library | Fractions derived from natural Chinese medicine |
|  | Size of the library | 10,000 natural compounds arrayed in 96-well plates as single compounds at 10 mM in DMSO |
| Library screened | Source | Pharmanic, China |
|  | Quality control | All compounds assured by the lab as > 90% pure on HPLC with QC data |
|  | Concentration tested | Constant 10 µM concentration, 0.1% DMSO for the initial screen; 8 concentration tested from 10 µM to 78.125 nM (half dilutions) for the validation screen |
|  | Format | 384-well plate |
|  | Plate controls | Positive control: peptide **3**; negative control: 5% DMSO |
|  | Plate number and duration | 250 plates over 30 days |
| Screen process | Reagent and compound dispensing systems | Biomek^®^ FX liquid handler (Beckman Coulter)  ZS-2 detector |
|  | Output, detector, analysis software | Fixed endpoint; FP value, SigmaPlot |
|  | Normalization | % inhibition = 100 x (sample result – average of positive control)/(average of negative – average of positive control) |
|  | Performance | Z’ = 0.68 (AdipoR1 system),  Z’ = 0.64 (AdipoR2 system) |
|  | Selection of actives | Actives were selected from the primary screen using a threshold of better than 5 µM (IC_50_) |
| Post-screen analysis | Retesting of initial actives | Original samples retested using screening assay condition; compounds with triplicated activity tested in dose-response mode (8 half dilutions) |
|  | List of validated compounds | Table **2** and Table **S2** |
